# 4070 Association of Interpersonal Processes of Care and Health Outcomes in Patients with Type II Diabetes

**DOI:** 10.1017/cts.2020.259

**Published:** 2020-07-29

**Authors:** Hadley Reid, Olivia M Lin, Rebecca L Fabbro, Kimberly S Johnson, Laura P. Svetkey, Bryan C Batch

**Affiliations:** 1 Duke University

## Abstract

OBJECTIVES/GOALS: 1. Understand the association between patient perceptions of care measured by the Interpersonal Processes of Care (IPC) Survey and glycemic control, appointment no-shows/cancellations and medication adherence in patients with type II diabetes. 2. Determine how these relationships differ by race for non-Hispanic White and Black patients. METHODS/STUDY POPULATION: This is a cross-sectional study of a random sample of 100 White and 100 Black Type II diabetic patients followed in Duke primary care clinics and prescribed antihyperglycemic medication. We will recruit through email and phone calls. Enrolled patients will complete the Interpersonal Processes of Care Short Form and Extent of Medication Adherence survey to measure patient perceptions of care (predictor) and medication adherence (secondary outcome). No show appointments and cancellations (secondary outcomes) and most recent hemoglobin A1c (primary outcome) will be collected from the Electronic Medical Record. We will also collect basic demographic information, insurance status, financial security, significant co-morbidities, and number and type (subcutaneous vs oral) of antihyperglycemic medications. RESULTS/ANTICIPATED RESULTS: -The study is powered to detect a 0.6% difference in HbA1c, our primary outcome, between high and low scorers on the Interpersonal Processes of Care subdomains. -We expect that higher patient scores in the positive domains of the IPC survey and lower DISCUSSION/SIGNIFICANCE OF IMPACT: This study will provide information to develop and implement targeted interventions to reduce racial and ethnic disparities in patients with Type II diabetes. We hope to gain information on potentially modifiable factors in patient-provider interactions that can be intervened upon to improve prevention and long-term outcomes in these populations.

